# 164. Antimicrobial Susceptibility of Urogenital and Extragenital *Neisseria gonorrhoeae* Isolates Among Men Who Have Sex with Men – SURRG and eGISP, 2018–2019

**DOI:** 10.1093/ofid/ofab466.164

**Published:** 2021-12-04

**Authors:** Laura Quilter, Sancta St Cyr, Jaeyoung Hong, Lenore Asbel, Ilene Bautista, Bonnie Carter, Yanick Casimir, Michael Denny, Melissa Ervin, Raquel Gomez, Alesia Harvey, Justin Holderman, Kimberly Johnson, Robert Kohn, Emily Learner, Kerry Mauk, Timothy William Menza, Christie Mettenbrink, William Nettleton, Karen Nicosia, Cau D Pham, Christopher Ried, Karen Schlanger, Annah Schneider, Olusegun O Soge, Irina Tabidze, Stephanie N Taylor, Winston Tilghman, Cindy Toler, Hillard Weinstock, Elizabeth Torrone

**Affiliations:** 1 Division of STD Prevention / Centers for Disease Control and Prevention, Atlanta, GA; 2 Centers for Disease Control and Prevention, Atlanta, GA; 3 Philadelphia Department of Public Health, Philadelphia, Pennsylvania; 4 Southern Nevada Health District, Las Vegas, Nevada; 5 Oakland County Health Division, Pontiac, Michigan; 6 Miami-Dade County Department of Health, Miami, Florida; 7 Hawaii State Department of Health, Honolulu, Hawaii; 8 Columbus Health Department, Columbus, Ohio; 9 Milwaukee Health Department, Milwaukee, Wisconsin; 10 New York City Department of Mental Health and Hygiene, New York City, New York; 11 San Francisco Department of Public Health, San Francisco, California; 12 Oregon Health Authority, Portland, OR; 13 Denver Health and Hospital Authority, Denver, Colorado; 14 Kalamazoo County Health and Community Services Department, Kalamazoo, Michigan; 15 Ohio Department of Health, Columbus, Ohio; 16 Orange County Health Care Agency, Santa Ana, California; 17 Minnesota Department of Health, St. Paul, Minnesota; 18 University of Washington, Seattle, Washington; 19 Chicago Department of Public Health, Chicago, Illinois; 20 Louisiana State University Health Sciences Center, New Orleans, Louisiana; 21 County of San Diego Health & Human Services Agency, San Diego, California; 22 Guilford County Public Health, Greensboro, North Carolina

## Abstract

**Background:**

Extragenital gonococcal infections are common among men who have sex with men (MSM); however, data comparing antimicrobial susceptibilities of urogenital and extragenital *Neisseria gonorrhoeae* isolates are limited. We investigated differences in gonococcal antimicrobial susceptibility by anatomic site among cisgender MSM using specimens collected through CDC’s enhanced Gonococcal Isolate Surveillance Project (eGISP) and Strengthening the U.S. Response to Resistant Gonorrhea (SURRG).

**Methods:**

During January 1, 2018–December 31, 2019, 12 eGISP and 8 SURRG sites collected urogenital, pharyngeal, and rectal isolates from cisgender MSM in STD clinics. Gonococcal isolates were sent to regional laboratories for antimicrobial susceptibility testing by agar dilution. To account for correlated observations, linear mixed-effects models were used to calculate geometric mean minimum inhibitory concentrations (MICs) and mixed-effects logistic regression models were used to calculate the proportion of isolates with elevated or resistant MICs; comparisons were made across anatomic sites.

**Results:**

Participating clinics collected 3,974 urethral, 1,553 rectal, and 1,049 pharyngeal isolates from 5,456 unique cisgender MSM. There were no significant differences in the geometric mean MICs for azithromycin, ciprofloxacin, penicillin, and tetracycline by anatomic site. For cefixime and ceftriaxone, geometric mean MICs for pharyngeal isolates were higher compared to anogenital isolates (p< 0.05). The proportion of isolates with elevated ceftriaxone MICs (≥ 0.125 µg/ml) at the pharynx (0.67%) was higher than at rectal (0.13%) and urethral (0.18%) sites (p< 0.05).

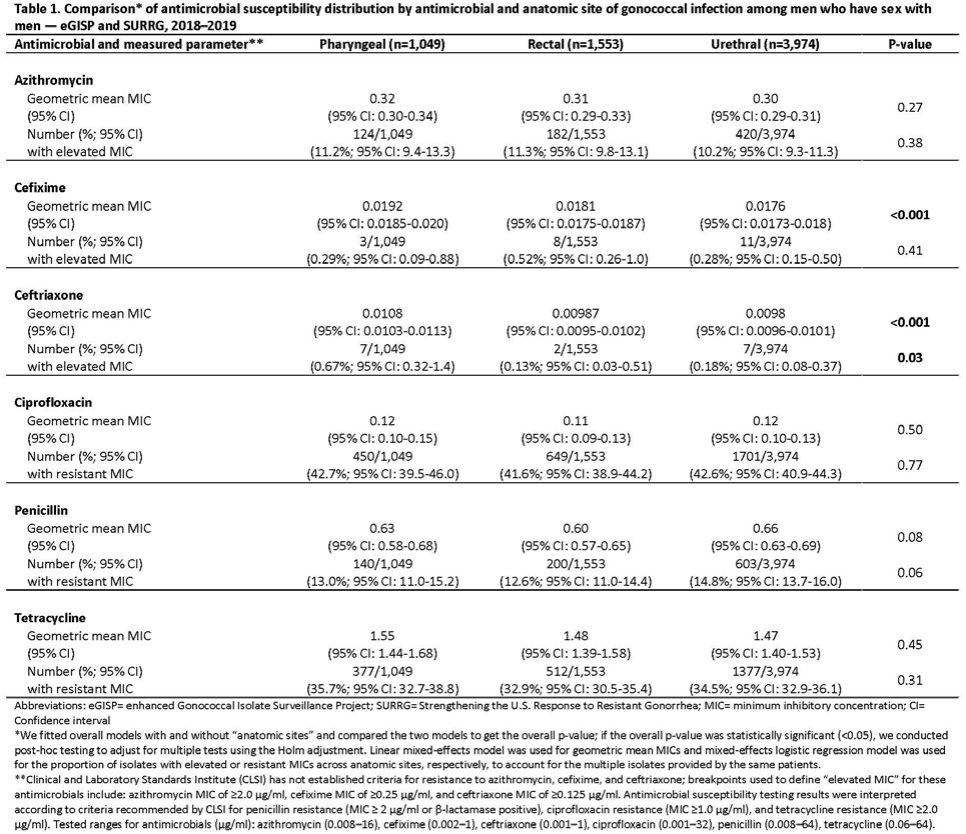

**Conclusion:**

Based on data collected from multi-jurisdictional sentinel surveillance projects, antimicrobial susceptibility patterns of *N. gonorrhoeae* isolates may differ among MSM at extragenital sites, particularly at the pharynx. Continued investigation into gonococcal susceptibility patterns by anatomic site may be an important strategy to monitor and detect the emergence of antimicrobial resistant gonorrhea over time.

**Disclosures:**

**Olusegun O. Soge, PhD**, **Hologic Inc.** (Grant/Research Support)**SpeeDx Inc.** (Grant/Research Support) **Stephanie N. Taylor, MD**, **GARDP - GC Antibiotic Development** (Scientific Research Study Investigator, To my institution.)**GlaxoSmithKline** (Grant/Research Support, Funds to my institution.)

